# Comparing Equil patch versus traditional catheter insulin pump in type 2 diabetes using continuous glucose monitoring metrics and profiles

**DOI:** 10.1111/1753-0407.13536

**Published:** 2024-04-10

**Authors:** Yu‐Jiao Li, Zi‐Yue Shao, Yun‐Qing Zhu, Da‐Shuang Chen, Jian Zhu

**Affiliations:** ^1^ Department of Endocrinology, Nanjing First Hospital Nanjing Medical University Nanjing China; ^2^ Department of Endocrinology Affiliated Hospital of Jiangnan University Wuxi China

**Keywords:** continuous glucose monitoring, patch insulin pump, T2DM

## Abstract

**Aims:**

It is not clear whether there are differences in glycemic control between the Equil patch and the MMT‐712 insulin pump. Our objective was to compare two types of insulin pumps in the treatment of type 2 diabetes mellitus (T2DM), using continuous glucose monitoring (CGM) metrics and profiles.

**Methods:**

This was a randomized case‐crossover clinical trial. Participants were hospitalized and randomly allocated to two groups and underwent two types of insulin pump treatments (group A: Equil patch—Medtronic MMT‐712 insulin pump; group B: Medtronic MMT‐712—Equil patch insulin pump) separated by a 1‐day washout period. Glycemic control was achieved after 7–8 days of insulin pump therapy. Each patient received CGM for 5 consecutive days (from day 1 to day 5). On day 3 of CGM performance, the Equil patch insulin pump treatment was switched to Medtronic MMT‐712 insulin pump treatment at the same basal and bolus insulin doses or vice versa. CGM metrics and profiles including glycemic variability (GV), time in range (TIR, 3.9–10.0 mmol/L), time below range (TBR, <3.9 mmol/L), time above range (TAR, >10.0 mmol/L), and postprandial glucose excursions, as well as incidence of hypoglycemia.

**Results:**

Forty‐six T2DM patients completed the study. There was no significant difference in parameters of daily GV and postprandial glucose excursions between the Equil patch insulin pump treatment and the Medtronic insulin pump treatment. Similarly, there was no between‐treatment difference in TIR, TBR, and TAR, as well as the incidence of hypoglycemia.

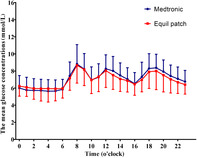

**Conclusion:**

The Equil patch insulin pump was similar to the traditional MMT‐712 insulin pump in terms of glycemic control. Equil patch insulin pump is a reliable tool for glycemic management of diabetes mellitus.

## Graphical abstract

Highlights

- This study was based on different parameters calculated from continuous glucose monitoring data and demonstrated that the Equil patch insulin pump has similar efficacy in clinical practice as the Medtronic MMT‐712 insulin pump.
- This study demonstrated that the Equil patch insulin pump had similar efficacy in controlling glucose variability, reducing hyperglycemia, and managing hypoglycemic episodes in type 2 diabetes mellitus patients as the Medtronic MMT‐712 insulin pump.

## 1 INTRODUCTION

Insulin pump therapy can control hyperglycemia and reduce glycemic variability, as well as improve the quality of life more effectively in both type 1 and type 2 diabetic patients than multiple daily insulin injections.^1^, ^2^, ^3^, ^4^, ^5^ Furthermore, early intensive insulin therapy using an insulin pump can improve islet beta cell function and induce glycemic remission in patients with newly diagnosed type 2 diabetes mellitus (T2DM).^6^, ^7^ Recent technologies have enabled significant advances in insulin pump design and functionality over the past decades. To date, two types of insulin pumps are commercially available: traditional and patch insulin pumps.^8^ In contrast to traditional insulin pumps, such as Medtronic, Roche, and Fornia, which administer insulin through a catheter inserted subcutaneously with tubing connecting the pump and cannula, Omnipod is a patch pump comprising a portable controller and a disposable pod that dispenses insulin.^9^ Despite the assertion of all insulin pump manufacturers regarding the conformity of their products with the international infusion pump standard EN 60601‐2‐24:1998, significant variations in the precision of insulin delivery were observed across different insulin pumps, and previous studies have demonstrated that patch insulin pumps exhibit lower delivery accuracy than traditional pumps.^10^, ^11^, ^12^, ^13^ In contrast, changing the position of a traditional insulin pump in relation to its catheter results in significant inaccuracy in basal insulin delivery, whereas a patch insulin pump performs with significantly less variation.^14^ Theoretically, the differences in insulin delivery between the two types of insulin pumps may lead to variations in glycemic control in a clinical setting.

Only one type of patch insulin pump (Equil Patch Insulin Pump; Microtech Medical [Hangzhou] Co., Ltd., Hangzhou, China) is currently available in China. The newly developed patch insulin pump comprises distinct pump and controller modules wherein the controller and display constituents are integrated into a wireless remote controller. Users can input operation commands on an LCD touch panel and adjust settings for insulin delivery via wireless communication. There have been studies on the efficacy of two pumps in patients with type 1 diabetes; however, it is not known whether the Equil patch insulin pump has the same efficacy and safety profile as the traditional catheter insulin pump in T2DM patients. Thus, we performed this randomized, case‐crossover study to compare Equil patch versus Traditional MMT‐712 insulin pump in type 2 diabetes using continuous glucose monitoring (CGM) metrics and profiles.

## 2 METHODS

### 2.1 Subjects and study design

This was a single‐center, randomized, case‐crossover clinical trial (Chinese Clinical Trial Register Identifier: ChiCTR2100043778). The study protocol was approved by the Institutional Ethics Committee of Nanjing First Hospital, Nanjing Medical University. Informed consent was obtained from all the participants. All procedures were performed in accordance with the Helsinki Declaration of 1964 as revised in 2013. From July 2020 to July 2021, inpatients with T2DM were recruited from the Department of Endocrinology of Nanjing First Hospital. The inclusion criteria were as follows: (a) age between 18 and 75 years; (b) body mass index ≥18 and ≤35 kg/m^2^; (c) glycosylated hemoglobin A1c (HbA1c) ≥ 8% in newly diagnosed patients and HbA1c ≥ 7% in those who had received hypoglycemic treatment. The exclusion criteria were similar to those in our previous study^15^: (a) type 1 diabetes, adult‐onset autoimmune diabetes mellitus, maturity‐onset diabetes in young, mitochondrial diabetes mellitus, antiglutamic acid decarboxylase antibody‐positive status, secondary diabetes mellitus, gestational diabetes mellitus, and stress hyperglycemia; (b) serious acute and/or chronic complications, including ketoacidosis or hyperosmolar state, severe cardiovascular disease, end‐stage renal disease, and severe infectious diseases; (c) treatment with weekly preparations of glucagon‐like peptide‐1 receptor agonists on admission; (d) known cancers; (e) pregnancy; and (f) cognitive disorders, alcoholism, or drug abuse.

The study design was similar to that used in our previous study.^15^ Participants were randomly allocated to two groups and underwent two trials separated by a 1‐day wash‐out period. Patients were randomly assigned to either group A (traditional MMT‐712 insulin pump‐Equil patch insulin pump) or group B (Equil patch insulin pump‐ Traditional MMT‐712 insulin pump). Insulin aspart (Novo Nordisk, Inc., Plainsboro, NJ) was used in insulin pumps during the study. Insulin doses were subsequently adjusted according to the patient's finger point blood glucose, which was monitored at seven time points: the pre‐ and 2 h‐postprandial of each meal, as well as the prebedtime. Glycemic control (fasting capillary blood glucose was<7.8 mmol/L and capillary blood glucose at 2 h after each of three meals was <10.0 mmol/L) was reached after a 7–8‐day run‐in period. A CGM System (Medtronic MiniMed, USA) was used to monitor the subcutaneous interstitial fluid glucose levels of the participants for 5 consecutive days. On day 3 of CGM, patients in group A switched to Traditional MMT‐712 insulin pump treatment at the same basic and premeal insulin doses or vice versa. Patients were instructed to maintain the same eating times, food volumes, and physical activity during the CGM study period.

The Abbott ARCHITECT I2000 automatic immunoanalyzer was used to analyze the biochemical results (Including fasting insulin and fasting C‐peptide, fasting blood glucose). We calculated homeostatic model assessment of insulin resistance (HOMA‐IR) and homeostatic model assessment of β‐cell function (HOMA‐β) by the formula HOMA‐IR = fasting glucose × fasting insulin ÷22.5 and the formula HOMA‐β = 20× fasting insulin ÷ (fasting glucose −3.5).^15^

During hospitalization, the caloric calories were calculated according to the following principles: diabetic meals were provided by the canteen, the total amount of fat, protein and carbohydrate were allocated according to the ratio of energy supply 3:2:5, and the calories were allocated according to the ratio of 1:2:2 for three meals. Patients were also asked to do 30–45 min of walking exercise between 15 min and 2 h after three meals a day during the hospital stay. The daily caloric intake and walking exercise remained stable during the study.

### 2.2 CGM

CGM data were obtained using the Medtronic Minimed CGM Gold system as previously described.^16^, ^17^, ^18^ The data collected from the CGM were from 0 am on day 2 to 0 am on day 3, and from 0 am on day 4 to 0 am on day 5. The CGM metrics and profiles were calculated as previously described,^16^, ^17^, ^18^ including 24‐h mean blood glucose (24‐h MBG), mean amplitude of glycemic excursion of blood glucose every 24 h (24‐h MAGE), SD of 24‐h blood glucose (24‐h SDBG), glucose coefficient of variation, time in range (TIR, 3.9–10.0 mmol/L), time below range (TBR, <3.9 mmol/L), time above range (TAR, >10.0 mmol/L), and glucose area under the curve (AUC) <3.9 mmol/L, between 3.9 mmol/L and 10.0 mmol/L, and >10.0 mmol/L, as well as postprandial glucose excursions, respectively. The postmeal relative area under the CGM curve (AUCpp) for 1–4 h was calculated as a measure of postprandial excursion. AUCpp was calculated as glucose excursion relative to the baseline (premeal) value. Postprandial glucose peaks were defined as the highest glucose value within a 3‐h window from the start of the meal.

**FIGURE 1:**
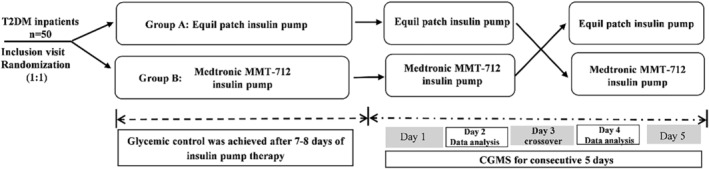
The study flow chart. CGMS, continuous glucose monitoring system; T2DM, type 2 diabetes mellitus.

All patients enrolled in our study were treated with insulin pump for 12–13 days, and the study design was described in the study flow chart (Figure 1).

### 2.3 Statistical analysis

Data were analyzed using the SPSS PASW Statistics 19 package, as described in our previous study.^16^ Normally distributed and continuous variables are presented as mean ± SD. Nonnormally distributed variables were presented as medians (interquartile ranges) and logarithmically transformed before analysis. The rates between the two groups were compared using the chi‐square test. Two‐way analysis of variance was used to compare the hourly glucose concentrations between the two groups. The crossover analysis was based on a univariate general linear model, including the treatment regimen and period as fixed effects and patients as a random effect. Statistical significance was set at *p* < .05.

## 3 RESULTS

### 3.1 Baseline characteristics of the participants

Fifty patients were enrolled in this clinical trial. Among these patients, three patients failed to reach the blood glucose standard after initial intensive treatment (including two patients in group A and one patient in group B) and one in group B was excluded due to inadequate CGM data. Finally, 46 subjects (92%) completed this study; the mean insulin dose was 0.55 ± 0.17 U/kg/day (Table 1). The proportion of newly diagnosed and nonnewly diagnosed patients in the two groups was not statistically significant by chi‐square test (*χ* ^2^ = 1.075, *p* = .300).

**TABLE 1:** The baseline characteristics of participants.

	ALL	Group A	Group B	*p* value
Sex (M/F)	46 (36/10)	23 (16/7)	23 (20/3)	.284
Age (years)	49.98 ± 12.31	49.91 ± 13.27	50.04 ± 11.57	.972
BMI (kg/m^2^)	25.12 ± 2.44	25.17 ± 0.21	25.06 ± 2.69	.884
Systolic BP (mmHg)	128.85 ± 14.92	130.30 ± 13.07	127.39 ± 16.73	.514
Diastolic BP (mmHg)	80.00 (80.00–90.00)	80.00 (74.00–86.00)	80.00 (80.00–90.00)	.299
ALT (U/L)	18.00 (12.00–29.00)	16.00 (10.50–29.50)	20.0 (13.75–28.25)	.343
AST (U/L)	12.00 (9.00–16.00)	12.00 (8.50–16.00)	1.50 (9.75–15.25)	.643
eGFR (mL/min/1.73 m^2^)	114.52 ± 17.45	112.44 ± 13.97	116.51 ± 20.36	.451
Total cholesterol (mmol/L)	5.35 ± 1.07	5.40 ± 1.26	5.31 ± 0.87	.773
Triglyceride (mmol/L)	1.74 (1.39–2.44)	1.83 (1.39–2.52)	1.69 (1.31–2.43)	.928
HDL‐cholesterol (mmol/L)	1.20 ± 0.23	1.22 ± 0.26	1.19 ± 0.21	.667
LDL‐cholesterol (mmol/L)	2.49 (2.08–3.15)	2.46 (2.19–3.20)	2.49 (2.01–2.75)	.578
FBG (mmol/L)	12.81 ± 2.66	13.29 ± 2.58	12.35 ± 2.71	.239
HbA1c (%)	11.03 ± 1.65	10.95 ± 1.55	11.11 ± 1.77	.753
HOMA‐β	10.69 (7.47–13.06)	8.17 (5.96–12.09)	11.22 (8.05–13.67)	.213
HOMA‐IR	2.70 (1.98–3.62)	2.92 (1.91–3.79)	2.38 (1.99–3.54)	.698
Fasting insulin (mIU/L)	4.60 (3.64–6.48)	4.65 (3.58–7.30)	4.60 (3.70–5.93)	.778
Fasting C‐peptide (pmol/l)	1.57 ± 0.49	1.65 ± 0.58	1.49 ± 0.37	.274
DID (IU/kg/day)	0.55 ± 0.17	0.59 ± 0.15	0.51 ± 0.18	.102
Basic insulin dosage (U/kg/day)	0.33 ± 0.12	0.36 ± 0.10	0.23 ± 0.08	.063
Preprandial insulin dosage (U/kg/day)	0.22 ± 0.09	0.30 ± 0.12	0.21 ± 0.09	.534

*Note*: Data are mean ± SD for normally distributed and continuous variables and median (interquartile range) for nonnormally distributed variables.

Abbreviations: ALT, alanine transaminase; AST, aspartate aminotransferase; BMI, body mass index; BP, blood pressure; DID, Daily insulin dosage; eGFR, estimated glomerular filtration rate; FBG, fasting plasma glucose; HbA1c, glycosylated hemoglobin; HDL, high‐density lipoprotein; HOMA‐IR, homeostatic model assessment of insulin resistance; HOMA‐β, homeostasis model assessment of pancreatic beta cell function; LDL, low‐density lipoprotein.

### 3.2 Glycemic variation profiles

The CGM glucose profiles of patients in each treatment group are shown in Table 2. There were no significant differences in 24‐h MBG, 24‐h MAGE, 24‐h SDBG, 24‐h maximum blood glucose (MAXBG), and 24‐h minimum blood glucose (MINBG) between the two types of insulin pump treatment. The TIR, TBR, and TAR during Equil patch insulin pump treatment were similar to those during Medtronic MMT‐712 insulin pump treatment. Similarly, there were no statistically significant differences between the two types of insulin pumps in AUCs of blood glucose <3.9 mmol/L, 3.9–10 mmol/L, and >10.0 mmol/L (Table 2). We further analyzed the postprandial glucose excursions in each patient. As shown in Table 3, the premeal glucose levels and postprandial peak glucose levels for each meal during the Equil patch insulin pump treatment were similar to those observed during the Medtronic MMT‐712 insulin pump treatment. The postmeal peak height and average time to peak for breakfast, lunch, and dinner were also similar between the two periods. No significant differences were found in AUCpp values 1–4 h after breakfast, lunch, or dinner between the two insulin pump treatments.

**TABLE 2:** The effects of two types of insulin pump on blood glucose variability.

	Equil patch insulin pump	Medtronic insulin pump	*F* value	*p* value
24‐h MBG	6.97 ± 0.72	7.04 ± 0.78	0.250	.620
24‐h MAGE (mmol/L)	3.59 (2.57–5.17)	3.35 (2.48–4.83)	0.003	.959
24‐h SDBG (mmol/L)	1.55 ± 0.55	1.64 ± 0.43	2.043	.160
LAGE (mmol/L)	6.20 (4.75–7.15)	6.55 (5.50–8.03)	4.198	.046
MAXBG (mmol/L)	10.91 ± 1.97	11.30 ± 1.88	3.019	.089
MINBG (mmol/L)	4.55 ± 0.80	4.41 ± 0.82	1.985	.166
24‐h MBG (mmol/L)	6.97 ± 0.73	7.04 ± 0.77	0.250	.620
AUC <3.9	0.00 (0.00–0.00)	0.00 (0.00–0.00)	2.478	.123
TBR (%)	0.00 (0.00–0.00)	0.00 (0.00–0.00)	2.770	.103
AUC 3.9–10	4490.00 (3503.054810.31)	4278.78 (3927.254880.56)	3.005	.090
TIR (%)	94.62 (89.52–10.00)	93.40 (87.88–98.70)	1.739	.194
AUC >10	37.63 (0.00105.56)	43.25 (0.00144.00)	0.145	.705
TAR (%)	4.69 (0.00–9.81)	4.86 (0.00–10.59)	0.036	.850

*Note*: Data are mean ± SD for normally distributed and continuous variables and median (interquartile range) for nonnormally distributed variables.

Abbreviations: AUC >10, incremental area under the curve of glucose concentrations >10.0 mmol/L (mmol/L per day); AUC<3.9: decremental area over the curve of glucose concentrations<3.9 mmol/L (mmol/l per day); AUC3.9–10, incremental area under the curve of glucose concentrations between 3.9 and 10 mmol/L (mmol/l per day); LAGE, large amplitude of glycemic excursion; MAGE, mean amplitude of glycemic excursion; MAXBG, maximum blood glucose; MBG, mean glucose concentration; MINBG, minimum blood glucose; SDBG, SD of the MBG; TAR, percentage of time spent on glucose concentrations above 10.0 mmol/L; TBR, percentage of the time spent on glucose concentrations<3.9 mmol/L; TIR, percentage of the time spent on glucose concentrations between 3.9 and 10 mmol/L.

**TABLE 3:** The effects of two types of insulin pump on postprandial glucose excursions.

	Equil patch	Medtronic	*F* value	*p* value
Breakfast				
Premeal (mmol/L)	5.80 ± 1.00	5.55 ± 1.14	1.002	.322
PPG peak(mmol/L)	9.67 ± 1.86	9.89 ± 2.06	2.195	.146
Peak height (mmol/L)	4.08 ± 1.90	4.50 ± 2.03	3.550	.066
Time to peak (min)	109.42 ± 41.90	113.79 ± 42.84	0.537	.468
AUCpp CGM (min×mmol/L)				
1 h	43.34 ± 33.44	54.96 ± 43.58	0.534	.469
2 h	199.25 (99.75315.89)	193.43 (115.13–285.57)	0.260	.612
3 h	380.28 ± 219.32	405.97 ± 211.63	1.707	.198
4 h	504.25 ± 290.04	553.51 ± 298.07	2.197	.145
Lunch				
Premeal (mmol/L)	6.70 ± 1.51	6.57 ± 1.52	0.303	.585
PPG peak (mmol/L)	9.23 ± 1.64	9.91 ± 1.92	2.964	.092
Peak height (mmol/L)	3.16 ± 1.58	3.92 ± 2.26	2.178	.147
Time to peak (min)	78.29 (53.05101.25)	85.50 (60.89–113.58)	0.946	.336
AUCpp CGM (min×mmol/L)				
1 h	63.70 ± 40.65	70.53 ± 32.62	0.667	.418
2 h	193.55 ± 128.18	243.94 ± 131.88	2.298	.137
3 h	292.16 ± 197.29	395.04 ± 243.35	3.371	.073
4 h	375.33 ± 256.24	506.28 ± 325.81	3.360	.074
Dinner				
Premeal (mmol/L)	6.42 ± 1.47	6.25 ± 1.23	0.455	.503
PPG peak (mmol/L)	9.09 ± 2.20	9.24 ± 1.66	0.104	.749
Peak height (mmol/L)	3.08 ± 2.00	3.36 ± 1.57	0.263	.610
Time to peak (min)	73.08 (53.61114.10)	93.67 (62.23–117.75)	0.675	.416
AUCpp CGM (min×mmol/L)				
1 h	61.23 ± 38.24	70.92 ± 36.81	1.649	.206
2 h	190.97 ± 129.70	228.59 ± 124.14	0.908	.346
3 h	252.08 (123.19460.21)	352.09 (234.96–514.63)	0.612	.438
4 h	407.48 ± 325.82	480.58 ± 265.22	0.997	.504

*Note*: Data are mean ± SD for normally distributed and continuous variables and median (interquartile range) for non‐normally distributed variables.

Abbreviations: AUCpp CGM, postmeal relative areas under the continuous glucose monitoring curve; peak height, calculated as (PPG peak—premeal glucose); PPG, postprandial glucose.

**FIGURE 2:**
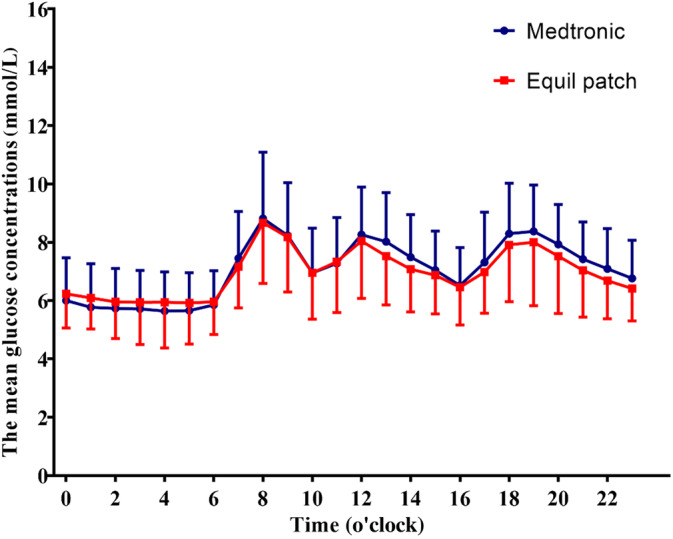
Comparison of average hourly blood glucose levels during treatment with two types of insulin pumps.

The average glucose concentration per hour carrying 24‐h period was similar between Equil patch and Medtronic MMT‐712 insulin pump treatment group (*p* > .05, Figure 2).

### 3.3 Hypoglycemic episodes

Hypoglycemic episodes were defined as those consistent with a blood glucose <3.9 mmol/L with or without significant clinical symptoms. A severe hypoglycemic event is defined as requiring assistance or other emergency measures to correct hypoglycemia. No severe hypoglycemic episodes occurred during the study period. The incidence of hypoglycemic episodes during Equil patch insulin pump treatment (7 episodes) was similar to that during Medtronic MMT‐712 insulin pump treatment (8 episodes) (χ^2^ = 0.536, *p* = .464). Overall, the Equil patch insulin pump treatment did not increase the incidence of hypoglycemic episodes compared with the Medtronic MMT‐712 insulin pump treatment. Moreover, the AUC and percentage of time when the blood glucose level was <3.9 mmol/L as detected via CGM did not differ significantly between the two pump treatments (Table 2).

## 4 DISCUSSION

Based on different parameters calculated from CGM data, the present study demonstrated that the Equil patch insulin pump has similar efficacy in clinical practice as the traditional MMT‐712 insulin pump, which has been shown to be effective and is widely used in clinical practice for patients with type 1 and type 2 diabetes, not only for controlling glucose variability but also for reducing hyperglycemia, as well as in managing hypoglycemic episodes in T2DM patients, indicating that the Equil patch insulin pump is a reliable tool for the glycemic management of diabetes mellitus.

Despite previous research indicating significant variations in insulin delivery accuracy between two types of insulin pump,^10^, ^11^, ^12^, ^13^, ^14^ a clinical study conducted on patients with type 1 diabetes revealed no discernible differences in HbA1c reduction between patch insulin pumps and traditional insulin pumps.^19^ Although the HbA1c has been widely regarded as the benchmark for evaluating long‐term glycemic control,^20^ mounting evidence have indicated that glycemic variability (GV), represented by the 24‐h mean amplitude of glycemic excursion (24‐h MAGE) calculated from CGM data, is an independent risk factor for diabetic complications, even at similar HbA1c level.^21^, ^22^, ^23^ The management of GV is an essential part of blood glucose control. Compared with HbA1c and structured seven‐point self‐monitoring of blood glucose, CGM has become the gold standard for glycemic control assessment because it provides more detailed information on the glucose profile of patients.^24^, ^25^ At the same time, insulin pump therapy has been accepted as an effective method to not only reduce hyperglycemia but also minimize GV by tailoring personalized 24‐h basal insulin infusion and on‐demand bolus insulin delivery.^26^ Although the Equil patch insulin pump is mainly available in China, it can be accessed through e‐commerce platforms for diabetic patients from all over the world at competitive prices. The present study, for the first time, compared the glucose‐lowering efficacy of the Equil patch insulin pump and traditional MMT‐712 insulin pump in patients with T2DM and demonstrated that these two types of insulin pumps have the same efficacy in controlling GV, including 24‐h MAGE, 24‐h SDBG, 24‐h MAXBG, and 24‐h MINBG (Table 2), as evidenced by 24‐h MBG and pre‐ and postprandial blood glucose for each meal at the same dose. Moreover, they showed similar effects in the management of hyperglycemia. Furthermore, there were no statistical differences between the two treatments in postprandial maximum blood glucose, time to peak, and AUCpp 1–4 h for each meal, indicating that the Equil patch insulin pump had the same efficacy in controlling postprandial glucose excursions as the traditional MMT‐712 insulin pump.

Hypoglycemia and the fear of hypoglycemia are major barriers to the use of antidiabetic medicines.^27^ Hypoglycemia has been strongly associated with increased risk of cardiovascular events and death.^27^, ^28^, ^29^ Using CGM, we were able to identify asymptomatic or nocturnal hypoglycemia during continuous subcutaneous insulin infusion therapy. The frequency of hypoglycemic episodes (blood glucose <3.9 mmol/L) was similar between the two treatments. Therefore, these two types of insulin pumps, using the same basal and bolus insulin doses, have the same clinical efficacy in the management of hyperglycemia, control of postprandial glucose excursion, and risk of hypoglycemia.

The present study had some limitations. First, this was a short‐term, single‐center clinical study. Experienced specialist nurses were responsible for the operation of the insulin pumps, including filling the reservoir, rewinding the pump, inserting the infusion set into the patient's body, setting the daily basal rate, and delivering the bolus during the insulin pump treatments. No alerts owing to catheter entanglement were observed during the use of the traditional MMT‐712 insulin pump. The advantage of the patch insulin pump in eliminating catheter entanglement, which affects glucose control, was not demonstrated in this study. Second, we did not test user perception, satisfaction, or preference for the Equil or Medtronic insulin pumps. These differences may affect the clinical efficacy and safety of the two types of insulin pumps.

## 5 CONCLUSIONS

In summary, compared to the traditional MMT‐712 insulin pump, the Equil patch insulin pump has similar clinical efficacy on MAGE, MBG, premeal blood glucose, and postprandial glucose excursions as well as hypoglycemic episodes in T2DM patients. The Equil patch insulin pump is a useful tool for T2DM management.

## AUTHOR CONTRIBUTIONS

Jian Zhu contributed to the conception and design of the study. Yu‐jiao Li, Zi‐yue Shao, Yun‐qing Zhu, and Da‐shuang Chen conducted the study and collected data. Yu‐jiao Li, Zi‐yue Shao, and Jian Zhu contributed to data analysis. Yu‐jiao Li, Zi‐yue Shao, and Jian Zhu contributed to manuscript writing. Jian Zhu provided final approval of the manuscript. All named authors meet the International Committee of Medical Journal Editors (ICMJE) criteria for authorship for this manuscript, take responsibility for the integrity of the work as a whole and gave final approval for the version to be published.

## FUNDING INFORMATION

This study and article processing charges were supported by the National Key R&D Program of China (No. 2018YFC1314100), and Medical Science and Technology Development Foundation, Nanjing Municipality Health Bureau (JQX12006).

## DISCLOSURES

The authors (Yu‐jiao Li, Zi‐yue Shao, Yun‐qing Zhu, Da‐shuang Chen, Jian Zhu) have no conflict of interest to declare.

## Data Availability

The data sets generated and/or analyzed during the current study are not publicly available but are available from the corresponding author on reasonable request.

## References

[1] Fisher S , Huang J , DuBord A , et al. Continuous subcutaneous infusion versus multiple daily injections of insulin for Pregestational diabetes in pregnancy: a systematic review and meta‐analysis. J Diabetes Sci Technol. 2023;2016:19322968231186626. 10.1177/19322968231186626 PMC10563519 37542367

[2] Blair J , McKay A , Ridyard C , et al. Continuous subcutaneous insulin infusion versus multiple daily injections in children and young people at diagnosis of type 1 diabetes: the SCIPI RCT. Health Technol Assess. 2018;22:1‐112. 10.3310/hta22420 PMC6119825 30109847

[3] Pala L , Dicembrini I , Mannucci E . Continuous subcutaneous insulin infusion vs modern multiple injection regimens in type 1 diabetes: an updated meta‐analysis of randomized clinical trials. Acta Diabetol. 2019;56:973‐980. 30945047 10.1007/s00592-019-01326-5

[4] Blevins T , Lane W , Rodbard D , et al. Glucose variability and time in range in type 2 diabetes treated with U‐500R by pump or injection: CGM findings from the VIVID study. Diabetes Technol Ther. 2021;23:51‐58. 32631081 10.1089/dia.2020.0030

[5] Grunberger G , Bhargava A , Ly T , et al. Human regular U‐500 insulin via continuous subcutaneous insulin infusion versus multiple daily injections in adults with type 2 diabetes: the VIVID study. Diabetes Obes Metab. 2020;22:434‐441. 31865633 10.1111/dom.13947 PMC7065168

[6] Retnakaran R , Pu J , Emery A , et al. Determinants of sustained stabilization of beta‐cell function following short‐term insulin therapy in type 2 diabetes. Nat Commun. 2023;14:4514. 37500612 10.1038/s41467-023-40287-w PMC10374648

[7] Weng J , Li Y , Xu W , et al. Effect of intensive insulin therapy on beta‐cell function and glycaemic control in patients with newly diagnosed type 2 diabetes: a multicentre randomised parallel‐group trial. Lancet. 2008;371:1753‐1760. 18502299 10.1016/S0140-6736(08)60762-X

[8] Lal R , Leelarathna L . Insulin Pumps. Diabetes Technol Ther. 2020;22:S17‐s31. 32069156 10.1089/dia.2020.2502 PMC7207015

[9] Ginsberg BH . Patch pumps for insulin. J Diabetes Sci Technol. 2019;13:27‐33. 30070604 10.1177/1932296818786513 PMC6313281

[10] Ziegler R , Waldenmaier D , Kamecke U , Mende J , Haug C , Freckmann G . Accuracy assessment of bolus and basal rate delivery of different insulin pump systems used in insulin pump therapy of children and adolescents. Pediatr Diabetes. 2020;21:649‐656. 32003490 10.1111/pedi.12993

[11] Freckmann G , Kamecke U , Waldenmaier D , Haug C , Ziegler R . Accuracy of bolus and basal rate delivery of different insulin pump systems. Diabetes Technol Ther. 2019;21:201‐208. 30901232 10.1089/dia.2018.0376 PMC6477586

[12] Jahn LG , Capurro JJ , Levy BL . Comparative dose accuracy of durable and patch insulin infusion pumps. J Diabetes Sci Technol. 2013;7:1011‐1020. 23911184 10.1177/193229681300700425 PMC3879767

[13] Laubner K , Singler E , Straetener J , Siegmund T , Päth G , Seufert J . Comparative dose accuracy of durable and patch insulin pumps under laboratory conditions. Diabetes Technol Ther. 2019;21:371‐378. 31149838 10.1089/dia.2019.0089

[14] Zisser HC , Bevier W , Dassau E , Jovanovic L . Siphon effects on continuous subcutaneous insulin infusion pump delivery performance. J Diabetes Sci Technol. 2010;4:98‐103. 20167172 10.1177/193229681000400112 PMC2825629

[15] Matthews DR , Hosker JP , Rudenski AS , Naylor BA , Treacher DF , Turner RC . Homeostasis model assessment: insulin resistance and beta‐cell function from fasting plasma glucose and insulin concentrations in man. Diabetologia. 1985;28(7):412‐419. 3899825 10.1007/BF00280883

[16] Luo Y , Wang XQ , Ni WJ , et al. Comparison of efficacy and economic value of Prandilin 25 and Humalog mix 25 in patients with newly diagnosed type 2 diabetes by a continuous glucose monitoring system. Diabetes Ther. 2018;9:2219‐2228. 30244319 10.1007/s13300-018-0502-5 PMC6250620

[17] Zhu J , Yuan L , Ni WJ , Luo Y , Ma JH . Association of Higher Circulating Insulin Antibody with increased mean amplitude glycemic excursion in patients with type 2 diabetes mellitus: a cross‐sectional. Retrospective Case‐Control Study J Diabetes Res. 2019;2019:7304140. 31687408 10.1155/2019/7304140 PMC6800966

[18] Luo Y , Ni WJ , Ding BO , et al. Efficacy comparison of Preprandial and postprandial Prandilin 25 Administration in Patients with newly diagnosed type 2 diabetes using a continuous glucose monitoring system. Diabetes Ther. 2019;10:205‐213. 30610472 10.1007/s13300-018-0545-7 PMC6349270

[19] Leelarathna L , Roberts SA , Hindle A , et al. Comparison of different insulin pump makes under routine care conditions in adults with type 1 diabetes. Diabet Med. 2017;34:1372‐1379. 28636773 10.1111/dme.13412

[20] Targets G . Standards of medical Care in Diabetes‐2020. Diabetes Care. 2020;43:S66‐s76. 31862749 10.2337/dc20-S006

[21] Monnier L , Owens D , Colette C , Bonnet F . Glycaemic variabilities: key questions in pursuit of clarity. Diabetes Metab. 2021;47:101283. 34547451 10.1016/j.diabet.2021.101283

[22] Brownlee M , Hirsch IB . Glycemic variability: a hemoglobin A1c‐independent risk factor for diabetic complications. Jama. 2006;295:1707‐1708. 16609094 10.1001/jama.295.14.1707

[23] Costantino S , Paneni F , Battista R , et al. Impact of glycemic variability on chromatin remodeling, oxidative stress, and endothelial dysfunction in patients with type 2 diabetes and with target HbA(1c) levels. Diabetes. 2017;66:2472‐2482. 28634176 10.2337/db17-0294

[24] Lee I , Probst D , Klonoff D , Sode K . Continuous glucose monitoring systems ‐ current status and future perspectives of the flagship technologies in biosensor research. Biosens Bioelectron. 2021;181:113054. 33775474 10.1016/j.bios.2021.113054

[25] Tian T , Aaron RE , Yeung AM , et al. Use of continuous glucose monitors in the hospital: the diabetes technology society hospital meeting report 2023. J Diabetes Sci Technol. 2023;17:1392‐1418. 37559371 10.1177/19322968231186575 PMC10563530

[26] Freckmann G , Buck S , Waldenmaier D , et al. Insulin pump therapy for patients with type 2 diabetes mellitus: evidence, current barriers, and new technologies. J Diabetes Sci Technol. 2021;15:901‐915. 32476471 10.1177/1932296820928100 PMC8258526

[27] Nakhleh A , Shehadeh N . Hypoglycemia in diabetes: an update on pathophysiology, treatment, and prevention. World J Diabetes. 2021;12:2036‐2049. 35047118 10.4239/wjd.v12.i12.2036 PMC8696639

[28] Christou M , Christou P , Kyriakopoulos C , Christou G , Tigas S . Effects of hypoglycemia on cardiovascular function in patients with diabetes. Int J Mol Sci. 2023;24:24. 10.3390/ijms24119357 PMC10253702 37298308

[29] Diouri O , Cigler M , Vettoretti M , Mader J , Choudhary P , Renard E . Hypoglycaemia detection and prediction techniques: a systematic review on the latest developments. Diabetes Metab Res Rev. 2021;37:e3449. 33763974 10.1002/dmrr.3449 PMC8519027

